# SFRP4 Reduces Atherosclerosis Plaque Formation in ApoE Deficient Mice

**DOI:** 10.1155/2023/8302289

**Published:** 2023-04-25

**Authors:** Hua Guan, Ting Liu, Miaomiao Liu, Xue Wang, Tao Shi, Fengwei Guo

**Affiliations:** ^1^Laboratory Animal Center, Xi'an Jiaotong University Health Science Center, Xi'an, Shaanxi 710061, China; ^2^Shaanxi Key Laboratory of Ischemic Cardiovascular Diseases & Institute of Basic and Translational Medicine, Xi'an Medical University, Xi'an 710021, Shaanxi, China; ^3^Department of Nephrology, Xi'an People's Hospital (Xi'an Fourth Hospital), Xi'an 710004, Shaanxi, China; ^4^Department of Cardiovascular Surgery, The First Affiliated Hospital of Xi'an Jiaotong University, Xi'an 710061, Shaanxi, China

## Abstract

Secreted frizzled related protein 4 (SFRP4), a member of the SFRPs family, contributes to a significant function in metabolic and cardiovascular diseases. However, there is not enough evidence to prove the antiatherosclerosis effect of SFRP4 in ApoE knock-out (KO) mice. ApoE KO mice were fed a western diet and injected adenovirus (Ad)-SFRP4 through the tail vein for 12 weeks. Contrasted with the control cohort, the area of atherosclerotic plaque in ApoE KO mice overexpressing SFRP4 was reduced significantly. Plasma high-density lipoprotein cholesterol was elevated in the Ad-SFRP4 group. RNA sequence analysis indicated that there were 96 differentially expressed genes enriched in 10 signaling pathways in the mRNA profile of aortic atherosclerosis lesions. The analysis data also revealed the expression of a number of genes linked to metabolism, organism system, and human disease. In summary, our data demonstrates that SFRP4 could play an important role in improving atherosclerotic plaque formation in the aorta.

## 1. Introduction

Cardiovascular disease (CVD) is the main cause of death in the world, causing 16.7 million deaths every year [1]. Most of CVD are caused by atherosclerosis, which is a disease characterized by the formation of plaque containing lipids and (immune) cells in the intima of large and medium-sized arteries [2]. In the process of atherosclerotic plaque formation, unstable atherosclerotic plaque rupture, vascular stenosis or occlusion caused by platelet aggregation, and thrombosis lead to acute CVD [3]. Atherosclerosis-related inflammation is mediated by proinflammatory cytokines, inflammatory signaling pathways, bioactive lipids, and adhesion molecules [4]. The most devastating consequences of atherosclerosis, such as heart attacks and strokes, are caused by superimposed thrombosis [5]. Therefore, atherosclerosis will be a more benign disease if we detect plaque prone to thrombosis and avoid thrombosis [6]. Therefore, it is necessary to further study and develop effective antiatherosclerosis and cardiovascular treatment strategies.

In 1997, Rattner et al. identified and described a new mammalian gene family, which encodes a secretory protein homologous to the cysteine-rich ligand binding region in the transmembrane receptor frizzled (Fz) family and is named secretory frizzled related proteins (SFRPs), which mediate the cell-cell signal connection of the Wnt signaling pathway [7]. The size of the SFRPs family proteins is about 30 kDa [8]. Each protein contains a signal peptide sequence, a coiled cysteine-rich domain (CRD), and a conserved hydrophilic carboxyl terminal domain [9]. Based on the structural characteristics of SFRP's protein, it not only combines with Wnt's protein to function but also antagonizes the activity of Wnt protein [7]. On the other hand, it can also bind with the FZ receptor and affect the direction of intracellular signal transduction [7]. As a member of the SFRPs family, since the discovery of SFRP4 [10], studies on its role in diabetes [11], obesity [12], and lipid metabolism [13–16] have continuously revealed its important roles, providing a new direction and strategy for the treatment of diabetes and lipid metabolism-related diseases. In addition, the latest research studies found that SFRP4 also plays an important role in the occurrence and development of cardiovascular diseases [17]. However, the exact molecular mechanism of its action and signal pathway activation still need to be further studied.

Based on these findings, this study aims to explore the underlying molecular mechanisms governing SFRP4 regulation of atherosclerosis. In order to confirm this assumption, ApoE knockout (KO) mice, the most popular animal model for human atherosclerosis, were fedwith a western diet and injected with an adenovirus (Ad)-SFRP4 or an Ad-green fluorescent protein (GFP) adenovirus through the tail vein for 12 weeks. After 12 weeks, lipid profiles, aortic atherosclerosis, and RNA sequence analysis of differential gene expression were evaluated in the aorta of the Ad-SFRP4 injected mice and their control counterparts (Ad-GFP injected). This study answers two main questions: (1) Does SFRP4 injection through the tail vein affect aortic atherosclerosis and plasma lipids? (2) If not, what is the associated molecular mechanism? The findings of this study demonstrate that overexpression of SFRP4 significantly inhibits aortic atherosclerosis through multiple signaling pathways except for inflammation and oxidative [18].

## 2. Materials and Methods

### 2.1. Animals and Diets

Eight-weeks-old male ApoE KO mice and wild-type C57BL/6J mice were obtained from the Vital River Company (Vital River Company, Beijing, China). In the experiment, 1 × 10^10^ plaque-forming units of Ad-SFRP4 or Ad-GFP (as a control) were introduced into the ApoE KO mice by injection at the tail vein. Pentobarbital sodium (150 mg/kg body weight) was injected intraperitoneally to euthanize the mice. All mice were nourished utilizing a western diet that contained 21% fat and 0.15% cholesterol. The diets for the mice were produced by Vital River Company (Vital River Company, Beijing, China). The mice were divided into two cohorts, each comprising fifteen animals. The mice were housed in an air-conditioned room for a cycle of 12 hours of light and 12 hours dark. Water and food were allowed *ad labium*. Approval of the animal experiment protocol was obtained from the Laboratory Animal Administration Committee of Xi'an Jiaotong University Health Science Center and performed as per the guidelines for Animal Experimentation of Xi'an Jiaotong University Health Science Center as well as the Guide for the Care and Use of Laboratory Animals published by the US National Institutes of Health (NIH Publication number 85-23, revised 2011).

### 2.2. Construction of the Adenoviral SFRP4 Vector and Infection of the HEK293 Cells

A recombinant adenoviral vector encoding SFRP4 (Ad-SFRP4) was constructed according to a previously published method [19, 20]. SFRP4 cDNA was subcloned into the adenoviral shuttle plasmid pAdTrack-CMV. Following sequence confirmation, the recombinant shuttle plasmid was transformed into the BJ5183 competent cell. The recombinant adenovirus was packaged and amplified in HEK293A cells. Following purification, the viral titer was detected by TCID50. An empty adenoviral vector (Ad-GFP) was constructed as a control.

### 2.3. Biochemical Analyses

Mice were fasted overnight, after which blood was drawn from the tail vein. The blood was mixed with EDTA and then centrifuged at 1,500 rpm for 10 min and a temperature of 4°C to get plasma. High-density lipoprotein cholesterol (HDL-C), low-density lipoprotein cholesterol (LDL-C), and plasma total cholesterol (TC) were analyzed utilizing commercial assay kits (BioSino Bio-Technology & Science Inc., Beijing, China) [21].

The plasma sample from each animal was examined in triplicate and measured as per the protocol of the manufacturer utilizing a Benchmark microplate reader (170-6750XTU, Bio-Rad, Veenendaal, Netherlands).

### 2.4. Quantification of Atherosclerotic Lesion

To quantify atherosclerosis, pentobarbital sodium (150 mg/kg) was injected intraperitoneally to euthanize the mice, and the aortic trees were opened up and stained using oil red O. Analysis of the *en* face lesion size was performed utilizing the image analysis system (WinRoof Mitani Co., Tokyo, Japan) [21, 22].

To perform a microscopic examination of atherosclerotic lesions, frozen cross-sections were incised at the level of the aortic root. Specifically, the analysis involved ten cross-sections from each mouse. The sections were then stained using oil red O and hematoxylin-eosin (H&E) in order to quantify the lesion area. The image analysis system (WinRoof Mitani Co., Tokyo, Japan) was employed to quantify the area stained with oil red O [23].

### 2.5. Extraction of Total RNA and Construction of cDNA Library

The extraction of total RNA from the aortas was conducted utilizing RNAzol (Takara, Tokyo, Japan). Nanodrop (Thermo, Rockford, IL, USA) was utilized to determine the RNA purity and concentration, while the Agilent 2100 Bioanalyzer (Agilent Technologies, Santa Clara, CA, USA) was utilized to verify integrity. Purification of mRNA from the total RNA was performed utilizing the NEBNext® Poly(A) mRNA magnetic isolation module. The library with an insert size of 400 bp was built utilizing a NEBNext UltraTM RNA Library Prep Kit adhering to the recommendation of the Illumina manufacturer [24]. The quality of the library was analyzed using the Agilent Bioanalyzer 2100 system. The index-coded samples were then clustered on a cBot Cluster Generation System utilizing the TruSeq PE Cluster Kit v4-cBot-HS (Illumina) [25]. The sequencing of the library was conducted on an Illumina HiSeq. 2500 platform utilizing 100 bp paired end reads (Illumina, San Diego, CA, USA).

### 2.6. Analysis of Differentially Expressed Genes (DEGs)

The Perl script was employed for trimming the reads with contaminated adapters, over 0.25% low-quality bases (Phred quality score <20), or over 10% Ns. Subsequently, alignment of clean reads with the mouse reference genome (GRCm38) was conducted utilizing TopHat. Quantification and normalization of gene expression were conducted by Cufflinks in RPKM (reads per million per kilo bases) [26]. The DESeq software was utilized to analyze DEGs through a comparison between the Ad-GFP and Ad-SFRP4 cohorts [27]. The false discovery rate (FDR) was employed to set the significant threshold for the *p*-value in various tests. The absolute values of FDR <0.05 and fold change ≥2 were used to determine the significance of gene expression. To perform pathway and functional enrichment analysis, the DEGs were charted into the Kyoto Encyclopedia of Genes and Genomes (KEGG) datasets, and a *p*-value of ≤0.05 was utilized to determine the significantly enriched KEGG terms.

### 2.7. qRT-PCR Analysis

qRT-PCRwas performed as previously described [14, 20]. Briefly, total RNA was isolated from the aortas of the ApoE KO mice by using the TRIzol Plus (Invitrogen, Carlsbad, CA, USA), and a SuperScript® III First-Strand Synthesis System (Invitrogen, Carlsbad, CA, USA) was utilized to synthesize cDNA. Then, the TaKaRa TP800 (TaKaRa Biology Inc., Shiga, Japan) was used to perform real-time PCR analysis. The fold-change in relative gene expression was calculated using the 2^−ΔΔCT^ method, using *β*-actin (for mRNAs) as internal controls. The sequence of PCR primers is illustrated in Table S1.

### 2.8. Western Blotting

Plasma samples were collected and subjected to western blotting. Briefly, 10 *μ*L plasma samples were fractionated on 10% SDS-polyacrylamide gels and then transferred to Sequi-Blot polyvinylidene fluoride membranes (Bio-Rad, Hercules, CA, USA). The membranes were incubated with each primary antibody (Ab) (anti-SFRP4 1 : 1,000 and anti-*β*-actin 1 : 1000) at 4°C overnight, as recommended in the manufacturer's instructions. After washing 3 times, they were incubated with horseradish peroxidase conjugated secondary Ab for 2 hr. Signals were detected using the Immobilon reagent (Millipore, Billerica, MA, USA) and visualized using an LAS-400 Lumino Image Analyzer (Fujifilm, Co., Tokyo). Visualized signal intensities were quantitatively analyzed using MultiGauge software (Bio-Rad, Hercules, CA, USA). All primary Abs were purchased from Cell Signaling Technology (Beverly, MA, USA).

### 2.9. Statistical Analysis

All the data are articulated as mean ± SEM. Statistical analyses were executed utilizing either Welch's *t*-test if the *p*-value was not equivalent or student's *t*-test with an equivalent *F*-value. The disparity between the two cohorts is judged to be statistically significant if *p* ≤ 0.05.

## 3. Results

### 3.1. Overexpression of SFRP4 in ApoE KO Mice

**Figure 1:**
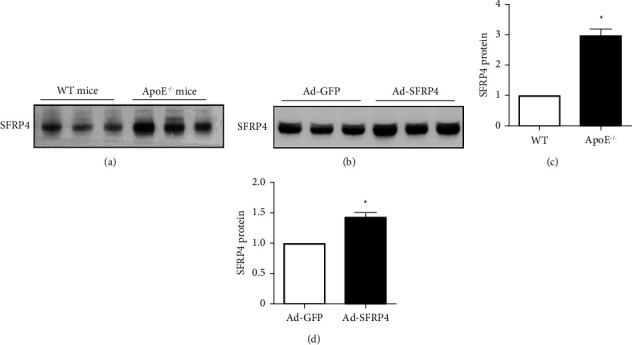
SFRP4 was overexpressed in ApoE KO mice. (a, b) Plasma protein expression of SFRP4 was ascertained by western blotting. (c, d) Quantification the western blotting. Data are expressed as the mean ± SEM. *n* = 6 of each cohort. ^*∗*^*p* < 0.05 versus Ad-GFP or WT. Ad-GFP, adenovirus-green fluorescent protein; KO, knock out; SFRP4, secreted frizzled related protein 4; WT, wild type.

To evaluate the protein expression differences between C57BL/6J and ApoE KO mice, plasma samples were determined by western blotting. It was demonstrated that SFRP4 protein expression was elevated in ApoE KO mice compared to wild type mice (Figure 1(a)). To determine if administering exogenous mouse SFRP4 has an effect on the formation of the atherosclerotic lesion, eight-weeks-old ApoE KO mice were treated systemically with adenoviral vectors expressing mouse SFRP4 (Ad-SFRP4) or control Ad-GFP. Circulating SFRP4 levels were approximately 1.5-fold higher in Ad-SFRP4 compared to Ad-GFP in ApoE KO mice six days after the systemic administration (Figure 1(b)). The outcomes displayed no significant bodyweight or food consumption differences between Ad-GFP and Ad-SFRP4 injected ApoE KO mice when they reached 20 weeks old (Figures S1A and S1B).

### 3.2. The Organ Weight and Plasma Parameters in ApoE KO Mice

After administrating Ad-SFRP4 for 12 weeks, we evaluated the organ weights of the liver, spleen, kidney, brown adipose tissue, inguinal white adipose tissue, and epididymis white adipose tissue. There was no significant difference in the organ weight after overexpression of SFRP4 compared to the GFP group (Figure S2).

**Figure 2:**
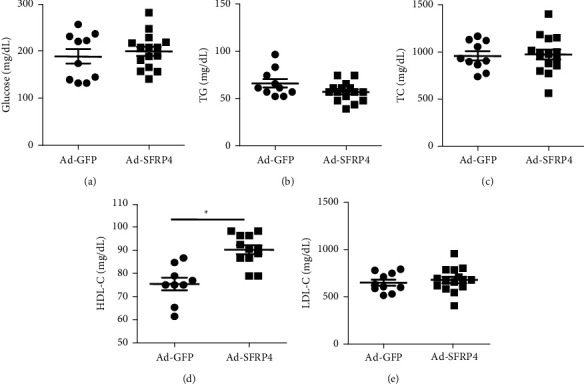
Plasma levels of total cholesterol (TC), low density lipoprotein cholesterol (LDL-C), and high density lipoprotein cholesterol (HDL-C). Data are expressed as the mean ± SEM. *n* = 10 for each group. ^*∗*^*p* < 0.05 Ad-SFRP4 versus Ad-GFP.

No significant differences were observed in metabolic parameters, such as triglyceride, total cholesterol, glucose, and LDL-C in the Ad-SFRP4 group compared to the Ad-GFP of ApoE KO mice (Figures 2(a)–2(c), and 2(e)). However, plasma HDL-C was significantly elevated after administered by Ad-SFRP4 compared to the GFP group (Figure 2(d)).

### 3.3. Increased Production of SFRP4 Reduced the Formation of Atherosclerotic Lesions in ApoE KO Mice

**Figure 3:**
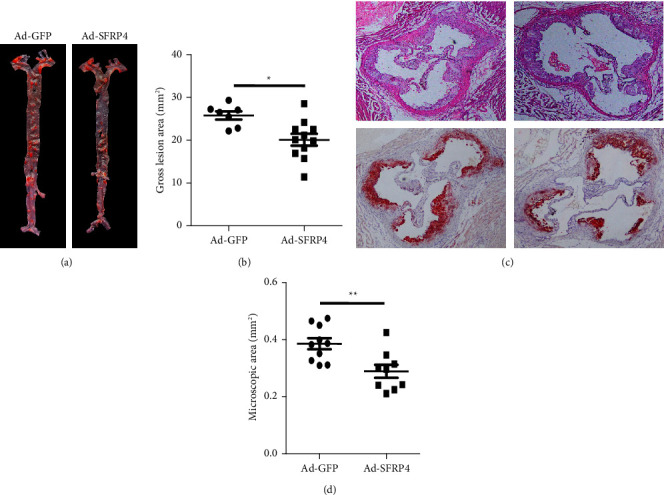
(a) Illustrative image of oil red O (ORO) staining in aortas. (b) The absolute value of gross lesion area. Illustrative micrographs of atherosclerotic lesions of the aortic root. (c) Aortic root sections stained using ORO and hematoxylin-eosin (H&E). (d) Quantitative analysis of aortic root lesion areas is illustrated at the right. Data are expressed as the mean ± SEM. *n* = 10 for each cohort. ^*∗*^*p* < 0.05Ad-SFRP4 versus Ad-GFP.

The size of the *en face* lesion in the total aorta was reduced considerably by 20% in the Ad-SFRP4 cohort as opposed to the control cohort (Figures 3(a) and 3(b)). The histological examination illustrated that aortic root atherosclerotic lesions also exhibited a decrease in the Ad-SFRP4 cohort. Microscopic initial lesions in the aortic root shrank considerably by 25% in the Ad-SFRP4 cohort as opposed to the control cohort (Figure 3(c)). Consequently, the lipid area in the lesions stained with oil red O was significantly shrunk by 20% in the Ad-SFRP4 cohort (Figure 3(d)).

### 3.4. Overexpression of SFRP4 Induces Genes Expression Antiatherosclerosis

**Figure 4:**
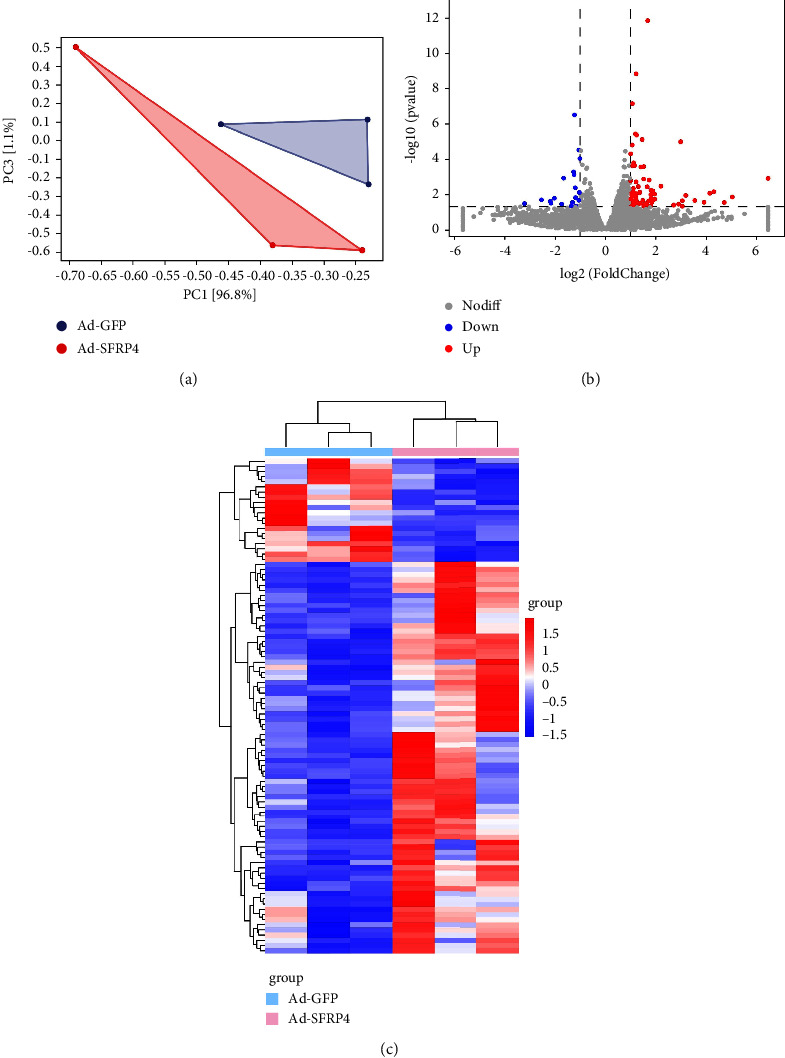
Typical characteristics of DEGs in the aorta of ApoE KO mice. (a) Principal components analysis (PCA) shows the difference of the samples. (b) Volcano plots show the DEGs expression based on the RNA sequences analysis. (c) Analysis of the DEGs was conducted utilizing the DESeq software by contrasting the Ad-GFP and Ad-SFRP4 cohort. The expression levels are denoted by colors, with red (high expression) and blue (low expression), and are proportional to their brightness (see color bar). *n* = 3 for each cohort.

Since atherosclerotic lesions were considerably smaller in the Ad-SFRP4 injection mice, altered gene expression levels in the lesions were investigated. For comparison, RNA sequence analysis was performed on aorta samples from Ad-SFRP4 and Ad-GFP-injected ApoE KO mice (Figure 4(a)). The transcriptomic analysis illustrated that there were 97 DEGs in the Ad-SFRP4 mice as opposed to the control mice. Among these DEGs, the upregulated genes were 77, while the downregulated genes were 20 (Figures 4(b) and 4(c), Table S2).

**Figure 5:**
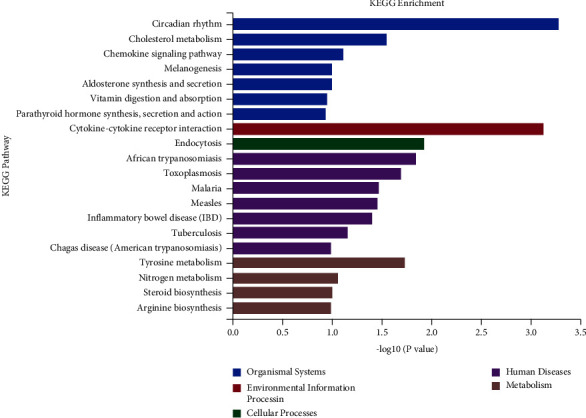
Enrichment KEGG pathways of DEGs in the aorta. The DEGs were charted into the KEGG datasets, significantly enriched KEGG terms were ascertained by *p* < 0.05. *n* = 3 for each cohort.

To investigate their functions, DEGs were grouped into five categories. The KEGG pathway analysis offered more potentially useful information illustrating the pathways pertinent to DEGs in atherogenesis. Based on our DEG outcomes, KEGG pathway analysis illustrated that these DEGs predominantly belonged to organism systems, environmental information, cellular processes, human diseases, and metabolism, such as cholesterol metabolism, chemokine signaling pathway, cytokine-cytokine receptor interaction, and nitrogen metabolism (Figure 5).

**Figure 6:**
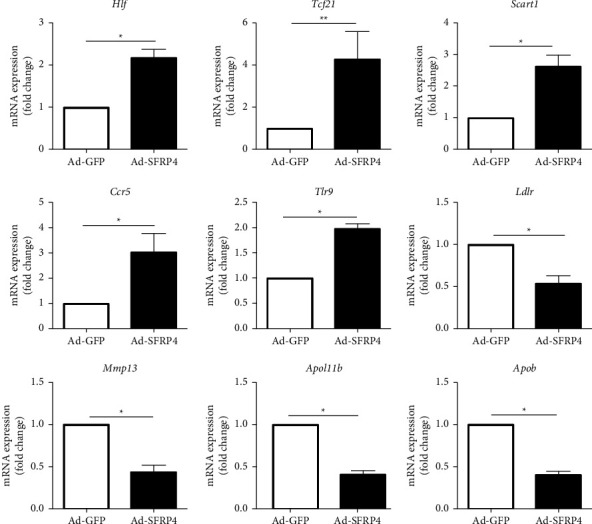
Real-time PCR was performed to identify gene expression in the RNAseq analysis. Genes were selected randomly in all 97 DEGs. *n* = 3 for each cohort. Data are articulated as mean ± SEM. ^*∗*^*p* < 0.05, Ad-SFRP4 versus Ad-GFP.

Moreover, qRT-PCR analysis of specific gene expression in the aorta, included *Hlf*, *Tcf21*, *Scart1*, *Ccr5*, *Tlr9*, *Ldlr*, *Mmp13*, *Apol11b*, and *Apob* yielded consistent results for the RNA sequence analysis (Figure 6).

## 4. Discussion

This study demonstrated that overexpression of SFRP4 inhibited aortic atherosclerosis in ApoE KO mice, however, no significant difference in plasma TC, TG, LDL-C, and glucose were observed, but only HDL-C was significantly elevated after overexpression of SFRP4, indicating that the practical effects of SFRP4 is partly dependent on increasing HDL-C plasma cholesterol levels [28]. This is a possibility that the achieved maximal effects are partly affected by liver lipid metabolism [29, 30]. Aortic lesions were considerably smaller in the SFRP4 overexpressing mice, and this was exemplified by a decreased accumulation of lipids. Reduced atherosclerotic lesions in the SFRP4 overexpressing cohort could result from multiple possible mechanisms. Hypothetically, the formation of foamy macrophages [31], the adhesion of monocytes to endothelial cells, and the synthesis and release of nitric oxide are vital points to reveal the potential molecular mechanisms contributing to the reduction of atherosclerosis [32, 33]. This contention was displayed by the outcomes of RNA sequence analysis, which suggested that multiple atherogenic genes, such as cholesterol metabolism, chemokine signaling pathways, and nitrogen metabolism [34–36]. Hence, it will be fascinating to examine whether SFRP4 has valuable effects on atherosclerosis.

Previous studies have shown that expression of SFRP4 in human ventricular myocardium correlates with apoptosis-related gene expression [37]. SFRP4 has been detected during cardiovascular maturation and in the adult heart. Moreover, knockdown of SFRP4 attenuates apoptosis to protect against myocardial ischemia/reperfusion injury [17]. Importantly, in our study, we found that the expression of SFRP4 was elevated in ApoE KO mice, indicating that SFRP4 play a role in the progression of atherosclerosis. To verify this hypothesis, we injected Ad-SFRP4 via the tail vein and found SFRP4 overexpression attenuated atherosclerosis plaque formation but also improved the plasma lipids, which was consistent with Zhang and his collegues [18]. In clinical research studies, SFRP4 is associated with impaired glucose and triglyceride metabolism in patients with stable coronary artery disease [38]. Compared to non-CAD patients, human epicardial adipose tissue-derived and circulating SFRP4 levels were increased in patients with CAD, indicating that the level of SFRP4 was independently associated with the presence of CAD [39]. To further confirm the protective effect of SFRP4 overexpression, RNA sequence analysis was performed to evaluate the underlying molecular mechanism about the inhabitation of atherosclerosis. These data demonstrated that overexpression of SFRP4 might have the potential to provide protection against atherosclerosis.

In this study, RNA sequence analysis of the aorta isolated from ApoE KO mice highlighted the DEGs enriched signaling pathways. SFRP4 is a unique and pleiotropic adipokine that has a protective function against the development of atherosclerosis via several mechanisms [18, 40]. Many clinical and epidemiological studies clearly show that HDL-C is negatively correlated with the risk of coronary heart disease (CHD), and it is a key and independent component to predict the risk of CHD [41]. The elucidation of HDL metabolism has produced therapeutic targets that may increase the level of plasma HDL-C, thereby reducing the risk of CHD [42]. The concept of reverse cholesterol transport is based on the assumption that HDL has a cardioprotective function, which is a process involving the removal of excess cholesterol accumulated by HDL in peripheral tissues, (such as macrophages in the aorta), transporting it to the liver, and excrete it into the feces through bile. Thereby, overexpression of SFRP4 activated the cholesterol metabolism signaling pathway and promoted the level of plasma HDL-C, which was consistent with the previous study [41]. Typically, *Ldlr*and *Apob-100* were downregulated significantly in Ad-SFRP4 group compared to the Ad-GFP group revealed that the formation of foamy macrophages was reduced by the decrease in uptake and transport of LDL [43].

As illustrated in Figure 5, we analyzed the percentage and number of DEGs in these signaling pathways. The outcomes illustrated that the cholesterol metabolism signaling pathway and cytokine-cytokine receptor interaction were the most extensive functional pathway, accounting for an aggregate of 9 DEGs (∼1% of the total). Existing evidence suggests that these enriched pathways are involved in cholesterol uptake and the ligand-receptor signal response in atherosclerosis [44, 45]. Our outcomes illustrated that Ad-SFRP4 mainly affects genes that participate in the formation of foamy macrophages in the aorta.

In conclusion, this study presents evidence that overexpression of SFRP4 protects against atherosclerosis. The underlying mechanism for the protective role of SFRP4 in the progress of plaque formation involves activation of cholesterol metabolism, nitric oxide synthesis, and chemokine signaling pathway. Therefore, circulating overexpression of SFRP4 may offer a potential effective for preventing atherosclerosis.

## Data Availability

The data used to support the findings of this study are available from the corresponding author upon reasonable request.

## Abbreviations

Ad: Adenovirus
CVD: Cardiovascular disease
CHD: Coronary heart disease
CRD: Cysteine rich domain
DEGs: Differentially expressed genes
FDR: False discovery rate
Fz: Frizzled
GFP: Green fluorescent protein
H&E: Hematoxylin-eosin
HDL-C: High-density lipoprotein cholesterol
KO: Knockout
KEGG: Kyoto encyclopedia of genes and genomes
LDL-C: Low-density lipoprotein cholesterol
SFRPs: Secretory frizzled related proteins
TC: Total cholesterol.

## Supplementary Materials

Supplementary Materials Supplementary Figure 1: Body weight and food consumption. (A) ApoE KO mice fed high fat and high cholesterol diet for 12 weeks and were evaluated the body weight and food consumption per mouse. *n* = 10 of each cohort. Data are expressed as the mean ± SEM. Supplementary Figure 2: The organs and adipose tissue weight. To evaluate the liver, kidney, spleen, brown adipose tissue (BAT), inguinal white adipose tissue (iWAT), epididymal white adipose tissue (eWAT). *n* = 10 of each corhort. Data are expressed as the mean ± SEM. Supplementary Table 1: Primers used for real-time PCR. Supplementary Table 2: Differentially expressed genes by RNA sequence analysis. RNA sequence analysis was performed on aorta samples from Ad-SFRP4 and Ad-GFP injected ApoE deficient mice for comparison. The transcriptomic analysis illustrated that there were 97 DEGs in the Ad-SFRP4 mice as opposed to the control mice. Among these DEGs, the upregulated genes were 77, while the downregulated genes were 20.
