# Supplementary figures and images for: SFRP4 Reduces Atherosclerosis Plaque Formation in ApoE Deficient Mice
Source: Cardiol Res Pract. 2023 Apr 25;2023:8302289. doi: 10.1155/2023/8302289 (PMC10154090; doi:10.1155/2023/8302289)

Supplementary Figure 1

A

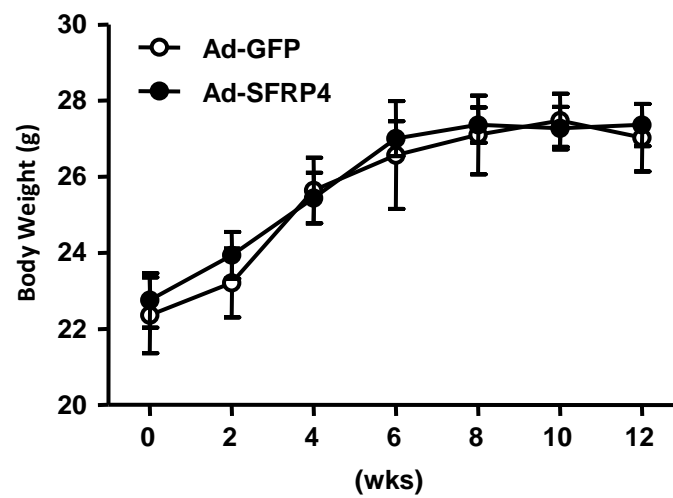

B

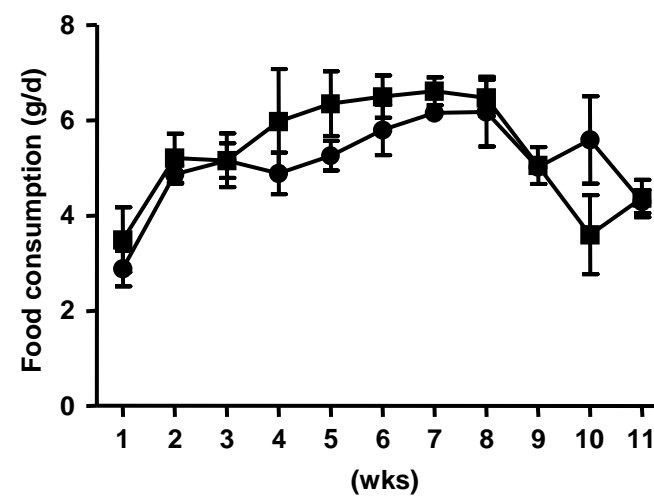

Supplementary Figure 2

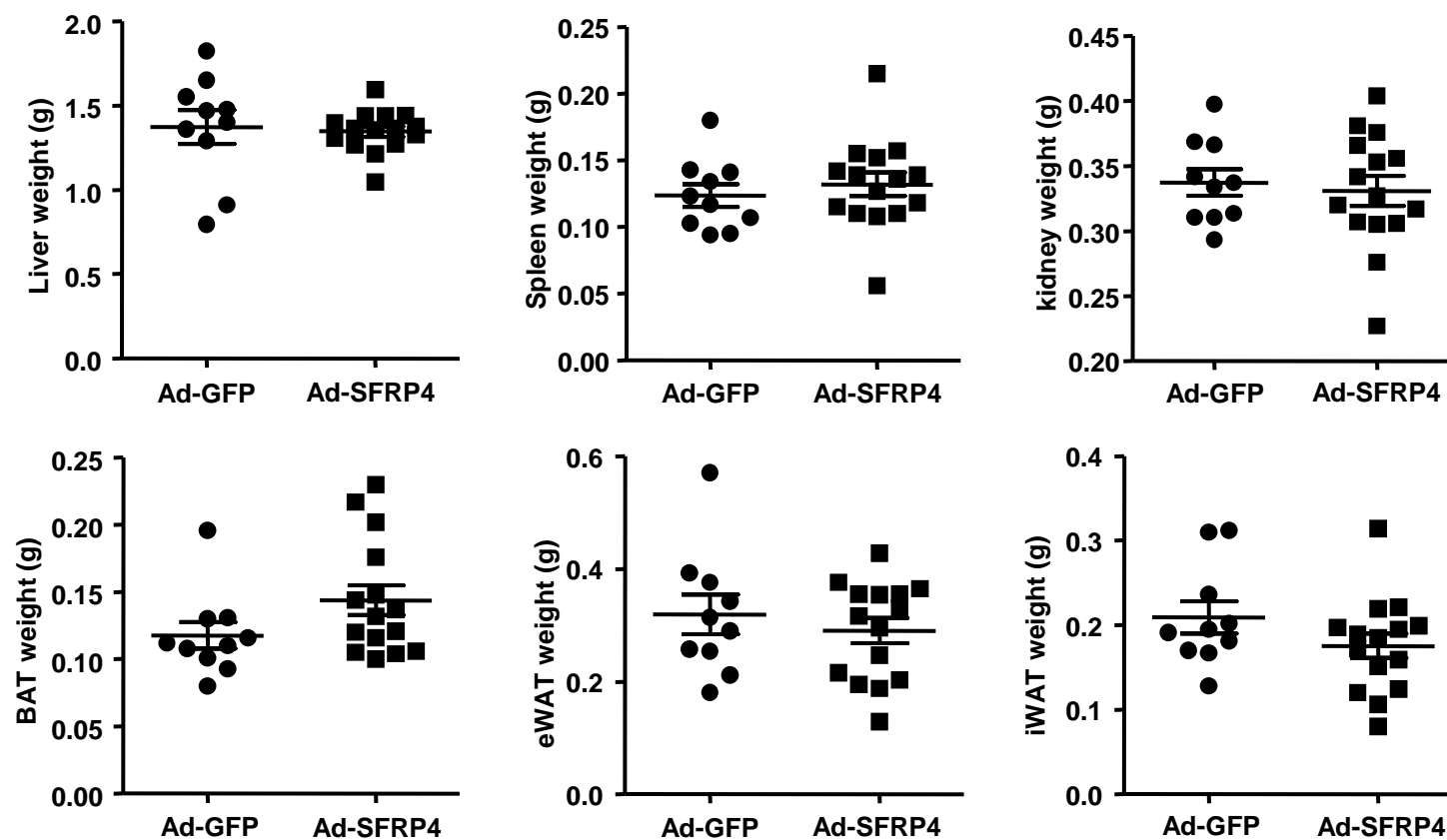

Supplement: Supplementary Materials — Supplementary Figure 1: Body weight and food consumption. (A) ApoE KO mice fed high fat and high cholesterol diet for 12 weeks and were evaluated the body weight and food consumption per mouse. n = 10 of each cohort. Data are expressed as the mean ± SEM. Supplementary Figure 2: The organs and adipose tissue weight. To evaluate the liver, kidney, spleen, brown adipose tissue (BAT), inguinal white adipose tissue (iWAT), epididymal white adipose tissue (eWAT). n = 10 of each corhort. Data are expressed as the mean ± SEM. Supplementary Table 1: Primers used for real-time PCR. Supplementary Table 2: Differentially expressed genes by RNA sequence analysis. RNA sequence analysis was performed on aorta samples from Ad-SFRP4 and Ad-GFP injected ApoE deficient mice for comparison. The transcriptomic analysis illustrated that there were 97 DEGs in the Ad-SFRP4 mice as opposed to the control mice. Among these DEGs, the upregulated genes were 77, while the downregulated genes were 20. [file 8302289.f1.zip › supplementary figure.pdf]
