# Supplementary material for: SFRP4 Reduces Atherosclerosis Plaque Formation in ApoE Deficient Mice
Source: Cardiol Res Pract. 2023 Apr 25;2023:8302289. doi: 10.1155/2023/8302289 (PMC10154090; doi:10.1155/2023/8302289)
Supplement: Supplementary Materials — Supplementary Figure 1: Body weight and food consumption. (A) ApoE KO mice fed high fat and high cholesterol diet for 12 weeks and were evaluated the body weight and food consumption per mouse. n = 10 of each cohort. Data are expressed as the mean ± SEM. Supplementary Figure 2: The organs and adipose tissue weight. To evaluate the liver, kidney, spleen, brown adipose tissue (BAT), inguinal white adipose tissue (iWAT), epididymal white adipose tissue (eWAT). n = 10 of each corhort. Data are expressed as the mean ± SEM. Supplementary Table 1: Primers used for real-time PCR. Supplementary Table 2: Differentially expressed genes by RNA sequence analysis. RNA sequence analysis was performed on aorta samples from Ad-SFRP4 and Ad-GFP injected ApoE deficient mice for comparison. The transcriptomic analysis illustrated that there were 97 DEGs in the Ad-SFRP4 mice as opposed to the control mice. Among these DEGs, the upregulated genes were 77, while the downregulated genes were 20. [file 8302289.f1.zip › Supplementary Table 1 Primer sequences of RT-PCR.doc]

**Supplementary Table 1.** Primers used for real-time RT-PCR

| Gene | Accession Number | Product  Length |  | Sequences（5’-3’） |
| --- | --- | --- | --- | --- |
| *Apob* | NM_009693 | 216 bp | Reveres | AGGTGGCCACAGCCAATAAT |
|  |  |  | Forward | CTTATGCCAGCTTGGTTGCAG |
| *Apol11b* | NM_001143686 | 232 bp | Reveres | AACAACACCCTGCTGCATCT |
|  |  |  | Forward | GGCCAGCAAATGATAAAGCCAA |
| *β-actin* | NM_007393.5 | 74 bp | Reveres | GCAGGAGTACGATGAGTCCG |
|  |  |  | Forward | ACGCAGCTCAGTAACAGTCC |
| *Hlf* | NM_001403985 | 183 bp | Reveres | CATCTGCAGCCGTCTACGTT |
|  |  |  | Forward | TTTCGTGTTCTCAGTGCCGA |
| *Ccr5* | NM_009917.5 | 187 bp | Reveres | GTTGTTTTGGAGAACGCCCC |
|  |  |  | Forward | CAACACTGCTCCGAAACTGC |
| *LDLr* | NM_001252658 | 109 bp | Reveres | CCAATCGACTCACGGGTTCA |
|  |  |  | Forward | ACAGTGTCGACTTCTCTAGGC |
| *MMP13* | NM_008607 | 189 bp | Reveres | AGAAGTGTGACCCAGCCCTA |
|  |  |  | Forward | GGTCACGGGATGGATGTTCA |
| *Scart1* | NM_172909 | 339 bp | Reveres | CGACCACTGCATTGTCTCCT |
|  |  |  | Forward | AGACTCCCGAGTGCCTATTCT |
| *Tcf21* | NM_011545 | 264 bp | Reveres | TTCACCCAGTCAACCTGACG |
|  |  |  | Forward | CGAATCTCCTCGTCGCAGTT |
| *Tlr9* | NM_031178 | 114 bp | Reveres | CTCCAACCGTATCCACCACC |
|  |  |  | Forward | GAGAAGTGCAGGGGGCTAAG |
