# Supplementary material for: SFRP4 Reduces Atherosclerosis Plaque Formation in ApoE Deficient Mice
Source: Cardiol Res Pract. 2023 Apr 25;2023:8302289. doi: 10.1155/2023/8302289 (PMC10154090; doi:10.1155/2023/8302289)
Supplement: Supplementary Materials — Supplementary Figure 1: Body weight and food consumption. (A) ApoE KO mice fed high fat and high cholesterol diet for 12 weeks and were evaluated the body weight and food consumption per mouse. n = 10 of each cohort. Data are expressed as the mean ± SEM. Supplementary Figure 2: The organs and adipose tissue weight. To evaluate the liver, kidney, spleen, brown adipose tissue (BAT), inguinal white adipose tissue (iWAT), epididymal white adipose tissue (eWAT). n = 10 of each corhort. Data are expressed as the mean ± SEM. Supplementary Table 1: Primers used for real-time PCR. Supplementary Table 2: Differentially expressed genes by RNA sequence analysis. RNA sequence analysis was performed on aorta samples from Ad-SFRP4 and Ad-GFP injected ApoE deficient mice for comparison. The transcriptomic analysis illustrated that there were 97 DEGs in the Ad-SFRP4 mice as opposed to the control mice. Among these DEGs, the upregulated genes were 77, while the downregulated genes were 20. [file 8302289.f1.zip › Supplementary Table 2 Differentially expressed genes.doc]

**Supplementary Table 2. Differentially expressed genes**

| **Name** | **Description** | **log2 Fold**  **Change** | ***p*-val** |
| --- | --- | --- | --- |
| Rgs20 | regulator of G-protein signaling 20 | -3.20 | 0.0335 |
| Tnr | tenascin R | -2.53 | 0.0205 |
| Tmem169 | transmembrane protein 169 | -2.19 | 0.0245 |
| Brinp1 | bone morphogenic protein/retinoic acid inducible neural specific 1 | -2.16 | 0.0318 |
| Cps1 | carbamoyl-phosphate synthetase 1 | -2.02 | 0.0166 |
| Gm19426 | predicted gene, 19426 | -1.73 | 0.0358 |
| Nfil3 | nuclear factor, interleukin 3, regulated | -1.66 | 0.0012 |
| Apob | apolipoprotein B | -1.35 | 0.0460 |
| Apol11b | apolipoprotein L 11b | -1.30 | 0.0287 |
| Mmp13 | matrix metallopeptidase 13 | -1.26 | 0.0005 |
| Dnajb1 | DnaJ heat shock protein family (Hsp40) member B1 | -1.24 | 0.0008 |
| Npas2 | neuronal PAS domain protein 2 | -1.22 | 0.0000 |
| Gpr176 | G protein-coupled receptor 176 | -1.19 | 0.0042 |
| Rab9b | RAB9B, member RAS oncogene family | -1.18 | 0.0153 |
| Nkx6-1 | NK6 homeobox 1 | -1.17 | 0.0156 |
| Wasf1 | WASP family, member 1 | -1.16 | 0.0469 |
| Nr4a2 | nuclear receptor subfamily 4, group A, member 2 | -1.05 | 0.0217 |
| Cyp51 | cytochrome P450, family 51 | -1.05 | 0.0000 |
| Arc | activity regulated cytoskeletal-associated protein | -1.03 | 0.0078 |
| Ldlr | low density lipoprotein receptor | -1.01 | 0.0001 |
| Gpr65 | G-protein coupled receptor 65 | 1.00 | 0.0284 |
| Siglec1 | sialic acid binding Ig-like lectin 1, sialoadhesin | 1.01 | 0.0017 |
| Ms4a6b | membrane-spanning 4-domains, subfamily A, member 6B | 1.01 | 0.0001 |
| Zim1 | zinc finger, imprinted 1 | 1.02 | 0.0183 |
| Klhl31 | kelch-like 31 | 1.02 | 0.0390 |
| Cilp | cartilage intermediate layer protein, nucleotide pyrophosphohydrolase | 1.05 | 0.0070 |
| Gpr34 | G protein-coupled receptor 34 | 1.05 | 0.0111 |
| Slc43a1 | solute carrier family 43, member 1 | 1.06 | 0.0087 |
| Rbm20 | RNA binding motif protein 20 | 1.07 | 0.0274 |
| Hlf | hepatic leukemia factor | 1.07 | 0.0000 |
| Zc3h6 | zinc finger CCCH type containing 6 | 1.07 | 0.0267 |
| Tef | thyrotroph embryonic factor | 1.08 | 0.0000 |
| Ms4a6c | membrane-spanning 4-domains, subfamily A, member 6C | 1.09 | 0.0002 |
| Adrb2 | adrenergic receptor, beta 2 | 1.10 | 0.0408 |
| Gm21188 | predicted gene, 21188 | 1.11 | 0.0072 |
| Ahsg | alpha-2-HS-glycoprotein | 1.11 | 0.0046 |
| Adgre1 | adhesion G protein-coupled receptor E1 | 1.12 | 0.0246 |
| Tlr9 | toll-like receptor 9 | 1.12 | 0.0125 |
| Clec4a1 | C-type lectin domain family 4, member a1 | 1.13 | 0.0002 |
| Msln | mesothelin | 1.14 | 0.0120 |
| Folr2 | folate receptor 2 (fetal) | 1.14 | 0.0392 |
| Mrc1 | mannose receptor, C type 1 | 1.16 | 0.0002 |
| Ccl19 | chemokine (C-C motif) ligand 19 | 1.19 | 0.0326 |
| Ccr5 | chemokine (C-C motif) receptor 5 | 1.20 | 0.0000 |
| Lyve1 | lymphatic vessel endothelial hyaluronan receptor 1 | 1.21 | 0.0091 |
| Il18r1 | interleukin 18 receptor 1 | 1.22 | 0.0167 |
| P2ry13 | purinergic receptor P2Y, G-protein coupled 13 | 1.22 | 0.0125 |
| Ms4a4a | membrane-spanning 4-domains, subfamily A, member 4A | 1.23 | 0.0020 |
| Nr1d2 | nuclear receptor subfamily 1, group D, member 2 | 1.23 | 0.0000 |
| Cybrd1 | cytochrome b reductase 1 | 1.23 | 0.0317 |
| Il1rl1 | interleukin 1 receptor-like 1 | 1.24 | 0.0230 |
| Hpgd | hydroxyprostaglandin dehydrogenase 15 (NAD) | 1.24 | 0.0000 |
| Extl1 | exostosin-like glycosyltransferase 1 | 1.28 | 0.0410 |
| Klhl33 | kelch-like 33 | 1.31 | 0.0036 |
| Mrgprb1 | MAS-related GPR, member B1 | 1.36 | 0.0082 |
| Muc16 | mucin 16 | 1.37 | 0.0074 |
| Slc14a1 | solute carrier family 14 (urea transporter), member 1 | 1.38 | 0.0321 |
| Tcap | titin-cap | 1.42 | 0.0003 |
| Per3 | period circadian clock 3 | 1.47 | 0.0000 |
| Trdn | triadin | 1.49 | 0.0215 |
| Ikzf3 | IKAROS family zinc finger 3 | 1.53 | 0.0014 |
| Scart1 | scavenger receptor family member expressed on T cells 1 | 1.53 | 0.0385 |
| Xirp2 | xin actin-binding repeat containing 2 | 1.53 | 0.0459 |
| Nrap | nebulin-related anchoring protein | 1.57 | 0.0500 |
| Zyg11a | zyg-11 family member A, cell cycle regulator | 1.63 | 0.0463 |
| Sbk2 | SH3-binding domain kinase family, member 2 | 1.65 | 0.0328 |
| Ciart | circadian associated repressor of transcription | 1.67 | 0.0037 |
| Nr1d1 | nuclear receptor subfamily 1, group D, member 1 | 1.69 | 0.0000 |
| Cgn | cingulin | 1.72 | 0.0233 |
| Il2ra | interleukin 2 receptor, alpha chain | 1.74 | 0.0016 |
| Asb11 | ankyrin repeat and SOCS box-containing 11 | 1.81 | 0.0445 |
| Cd163 | CD163 antigen | 1.81 | 0.0103 |
| Cxcr6 | chemokine (C-X-C motif) receptor 6 | 1.82 | 0.0058 |
| Fmo6 | flavin containing monooxygenase 6 | 1.83 | 0.0169 |
| Nlrp10 | NLR family, pyrin domain containing 10 | 1.85 | 0.0192 |
| Corin | corin, serine peptidase | 1.85 | 0.0281 |
| Tcf21 | transcription factor 21 | 1.89 | 0.0063 |
| Tph1 | tryptophan hydroxylase 1 | 1.94 | 0.0196 |
| Entpd4b | ectonucleoside triphosphate diphosphohydrolase 4B | 1.97 | 0.0186 |
| Asb15 | ankyrin repeat and SOCS box-containing 15 | 2.21 | 0.0034 |
| Sbk3 | SH3 domain binding kinase family, member 3 | 2.72 | 0.0407 |
| Slit1 | slit guidance ligand 1 | 2.72 | 0.0401 |
| Dnah3 | dynein, axonemal, heavy chain 3 | 2.92 | 0.0361 |
| Dbp | D site albumin promoter binding protein | 3.00 | 0.0000 |
| Gata4 | GATA binding protein 4 | 3.07 | 0.0228 |
| Gnrhr | gonadotropin releasing hormone receptor | 3.07 | 0.0475 |
| Epn3 | epsin 3 | 3.20 | 0.0115 |
| Trhde | TRH-degrading enzyme | 3.55 | 0.0221 |
| Dct | dopachrome tautomerase | 4.15 | 0.0086 |
| Cyp26a1 | cytochrome P450, family 26, subfamily a, polypeptide 1 | 4.31 | 0.0071 |
| BC051019 | cDNA sequence BC051019 | 4.73 | 0.0290 |
| Tyrp1 | tyrosinase-related protein 1 | 5.05 | 0.0141 |
| Il10 | interleukin 10 | Inf | 0.0013 |
